# The Association of Human Leukocyte Antigen Genotyping Among Sudanese Patients With Rheumatoid Arthritis: Reference to Ethnicity

**DOI:** 10.7759/cureus.43905

**Published:** 2023-08-22

**Authors:** Adil A Ali, Khalid E Khalid, Hajir M Hussien, Somaya E Mohammed, Osman K Saeed

**Affiliations:** 1 Department of Biochemistry and Nutrition, Faculty of Medicine, University of Gezira, Wad Medani, SDN; 2 Department of Basic Medical Sciences, Faculty of Applied Medical Sciences, Albaha University, Al-Baha, SAU; 3 Department of Medical Microbiology, Faculty of Medical Laboratory Sciences, University of Gezira, Wad Medani, SDN; 4 Department of Pathology, Faculty of Medicine, University of Gezira, Wad Medani, SDN; 5 Department of Internal Medicine, Faculty of Medicine, University of Gezira, Wad Medani, SDN

**Keywords:** human leukocyte antigen, rheumatoid factor, anti-ccp, hla-dqb1, hla-drb1, rheumatoid arthritis

## Abstract

Introduction: Human leukocyte antigens (HLA) account for up to one-half of the total genetic contribution to rheumatoid arthritis (RA) risk. The study investigated the association of HLA class II genotyping with RA susceptibility in Sudanese ethnic groups.

Methods: The DRB1 and DQB1 alleles and haplotypes were determined in 122 RA patients (i.e., Gaalia = 54, Johayna = 24, Baggara = 17, Nile Nubian = 12, and others = 15) and 120 healthy controls of ethnic groups (i.e., Gaalia = 44, Johayna = 11, Baggara = 15, Nile Nubian = 9, and others = 21) using a polymerase chain reaction with sequence-specific primers method.

Results: Susceptibility to RA was associated with a high frequency of DRB1*04 (P = 0.04), DRB1*10 (P = 0.04), and DQB1*03 (P = 2.2 x 10^-8^/P_c_ = 6.6 x 10^-8^) between study ethnic groups, while protective effects were shown with DRB1*07 (P = 0.01), DQB1*02 (P = 0.02), and DQB1*06 (P = 2.2 x 10^-6^/P_c_ = 6.6 x 10^-6^), with an inconsistent frequency between study ethnic groups. The HLA haplotypes that were high in frequency among RA ethnic groups and showed susceptibility associations were DRB1*03-DQB1*03, DRB1*04-DQB1*03, DRB1*08-DQB1*03, DRB1*13-DQB1*02, and DRB1*13-DQB1*03 (P = 0.00003/P_c_ = 0.0003, P = 0.0001/P_c_ = 0.0001, P = 0.03, P = 0.004/P_c_ = 0.03, and P = 3.79x10^-8^/P_c_ = 3.3x10^-9^, respectively). On the contrary, DRB1*03-DQB1*02, DRB1*07-DQB1*02, and DRB1*13-DQB1*06 were lower in frequency in the ethnic groups with RA and may confer protection (P = 0.004/P_c _= 0.032, P = 0.002/P_c_ = 0.02, and P = 0.01, respectively).

Conclusions: Our findings indicate an association between HLA-DRB1 and DQB1 genotypes and the susceptibility to RA in the Sudanese population, with a moderate frequency between our ethnic groups.

## Introduction

Rheumatoid arthritis (RA) is an autoimmune disease with features of chronic inflammation that affects synovial joints, leading to the destruction of joint cartilage [1]. RA pathogenesis is multifactorial, and disease susceptibility is associated with genetic and environmental factors [2].

The anti-citrullinated peptide antibodies (ACPA), which are more frequent than the rheumatoid factor (RF), present in 70-80% of RA patients, were considered predictive markers of bone destruction since they could be detected years before the disease and be stable after, and they were associated with disease erosion and aggressiveness [3,4]. Likewise, ACPA specificity is higher in RA than RF and was found to be strongly associated with human leukocyte antigen (HLA)-DRB1 shared epitope (SE) alleles such as DRB1*03 and *04, particularly in ACPA seropositive RA patients [2,5].

The HLA genes are situated on chromosome 6 (6p21.3), and their genomic map spans about 7.6 Mb and contains approximately 421 gene loci in a contiguous region on chromosome 6 [6]. Among the HLA genes, HLA-DRB1 SE alleles that encode for a common amino acid sequence are the strongest genetic risk factors for RA [7,8].

The expression of HLA class II, particularly the HLA-DRB1/DQB1 genotypes in RA, varies between racial groups and is geographically distributed from one population to another [9]. It has been reported that patients who carry HLA-DRB1 alleles are at high risk for developing a more progressive type of disease in many populations [10-12], including Sub-Saharan African populations [13,14]. Therefore, the genetic risk of reclassified HLA-DRB1/DQB1 alleles may vary among different ancestries, including our population, and raises the possibility that the specific HLA-DRB1/DQB1 alleles responsible for disease risk may differ in African and European ancestral populations [8].

Studies linking HLA-DRB1/DQB1 haplotypes and their association with RA susceptibility in the variable ethnic groups in our population are inadequately studied. Thus, investigating the allele frequency patterns of HLA genes that contribute to the development of RA in our population can provide insight into the disease's nature, vulnerability, and prognosis. This study relates HLA class II genotypes to RA vulnerability among our different ethnic populations.

## Materials and methods

Patients and controls

This is a hospital-based case-control study that retrospectively encompassed 122 RA patients (16 males with a mean age of 44.81 ± 14.65 years and 106 females with a mean age of 44.97 ± 14.01 years) and 120 healthy controls (11 males with a mean age of 38.55 ± 14.37 years and 89 females with a mean age of 43.62.55 ± 9.89 years).

The study was conducted in two different rheumatology outpatient clinics at the Academy Teaching Hospital and Ibrahim Malik Hospital in Khartoum, Sudan.

Sudan is a large country located in the northeast (i.e., the heart) of the African continent and shares borders with seven countries. Sudan's population structure is rooted in many diverse cultural, social, ethnic, verbal, and religious characteristics.

The patients were selected directly or indirectly from referrals to the clinics based on the clinical and laboratory examinations conducted under the supervision of two rheumatologists from each clinic. The RA diagnosis was assessed based on the criteria established in 2010 by the American College of Rheumatology [15].

The laboratory examinations were conducted accordingly and recorded together with the demographic and clinical findings on a well-structured questionnaire.

The healthy controls were selected based on the fact that they are apparently healthy, have no family history of RA, have a current rheumatic picture, or have any other autoimmune disease, and are recruited from the same hospitals and geographical areas. We considered the self-reports for the determination of age, sex, and ethnicity. Study subjects were categorized into 11 ethnic groups extracted from the Sudanese official documents of tribes and family histories as follows: Gaalia (44.3% vs. 44.0%), Johayna (19.7% vs. 11.0%), Baggara (13.9% vs. 15.0%), Nile Nubian (9.8% vs. 9.0%), Nubian (1.6% vs. 8.0%), Darfurian (0.8% vs. 5.0%), and Moroccan and Egyptian (3.3%). The Nubian, Darfurian, Moroccan, Egyptian, Nigerian, Beja, Fonj, and Chadian tribes were collectively named "others" because of their low frequency among study subjects.

Verbal and written informed consent was obtained from all the study participants. The study was approved by the Institutional Ethics Committee of the University of Gezira (approval number: UGIREC/2020).

HLA genotyping

DNA was extracted from the peripheral blood according to the manufacturer’s instructions provided by the QIA amp® DNA mini kit (Qiagen, Germantown, MD). Low-resolution kits (HLA-SSP Typing Kits, R.O.S.E. Europe GmbH, Frankfurt, Germany) for HLA class II typing were used with sequence-specific primers for a polymerase chain reaction (PCR-SSP). DNA amplification was done using an Applied Biosystem 9700 thermocycler (Thermo Fisher Scientific, Waltham, MA), and the protocol was applied consistently with the instructions provided by the company. The amplified DNA fragments were separated using 2% agarose gel electrophoresis and visualized under ultraviolet light. The typing of HLA genotypes was achieved via HLA software (R.O.S.E. Europe GmbH, Frankfurt, Germany).

Autoantibody analysis

Peripheral blood samples were collected from each study participant in vacuum tubes containing ethylenediaminetetraacetic acid (EDTA) and processed to obtain serum under good laboratory practice standards. Serum ACPA was detected using anti-cyclic citrullinated peptides (anti-CCP) IgG antibodies and was quantitatively and semiquantitatively detected as per the instructions provided in the Immunoscan CCPlus® ELISA kits (Euro Diagnostica AB, Malmö, Sweden). Positive results for ACPA were defined as concentrations >25 U/ml. A rapid latex agglutination test (NS Biotec, Alexandria, Egypt) and an automated chemistry analyzer (Cobas Mira Plus, Roche, Basel, Switzerland) were used to measure serum C-reactive proteins (CRP) and IgM RF, respectively. Erythrocyte sedimentation rate (ESR) measurement was performed using both capillary tube (micro ESR) and conventional Westergren methods simultaneously [16].

Statistical analysis

The sample size was calculated based on a prevalence rate of 0.5% and a power of study of 80% using G*Power 3.1.9.6 for MacOS X. The mean differences of quantitative variables between study subjects were compared by the independent sample t-test. All allele and haplotype frequencies were given as percentages. The chi-square test, or Fisher’s exact test, was used to compare the distribution of the HLA allele frequencies between study subjects. The direct counting and precise combination of alleles were used to determine the two-locus associations and haplotype frequencies (HF), while the chi-square test with Yates’ correction was used to determine the significance of associations. The strength of the associations was estimated by using the odds ratio (OR) and 95% confidence interval (CI). The Bonferroni correction was used for allele and haplotype groups with frequencies >5% in either cases or controls and applied when the P-values were 0.05. Corrected probability values (Pc) were determined by multiplying the individual P-values by the number of comparisons made at the allele or haplotype levels. SPSS version 23 (IBM Corp., Armonk, NY) was used for analysis, and P-values < 0.05 were considered significant.

## Results

Patients and healthy controls

The study included 122 RA patients, and their demographic and clinical findings are presented in Table 1. The mean levels of ACPA, CRP, and ESR were remarkably higher in RA patients compared with healthy controls. Gaalia was the most frequent tribe, accounting for 54 (44.3%) patients, followed by Johayna (24; 19.7%), Baggara (17; 13.9%), and Nile Nubian (12; 9.8%). Other tribes were collected together only because their numbers were too small for meaningful comparisons.

**Table 1:** Characteristics of RA patients and controls. * p = <0.05; n, number; NA, not available; %, percentage; SD, standard deviation; ACPA: anti-citrullinated peptide antibodies; CRP: C-reactive protein; ESR: erythrocyte sedimentation rate; RA: rheumatoid arthritis.

Parameters				RA patients (n = 122)	Controls (n = 100)
Sex (%)		Female		106 (86.9)	89 (89.0)
		Male		16 (13.1)	11 (11.0)
Mean age in years ±SD				44.95 ± 14.03	43.06 ± 10.51
Mean disease duration in years ±SD				3.56 ± 4.36	NA
ACPA (U/ml) (mean ± SD)				594.11 ± 80.42	25.00 ± 0.00*
CRP (mg/L) (mean± SD)				16.31 ± 1.83	1.9 ± 0.31*
ESR (mm/hr) (mean ± SD)				66.02 ± 2.62	28.59 ± 0.97*
Tribes (%)	Gaalia			54 (44.3)	44 (44)
	Johayna			24 (19.7)	11 (11)
	Baggara			17 (13.9)	15 (15)
	Nile Nubian			12 (9.8)	9 (9)
	Others		Nubian	2 (1.6)	8 (8)
			Darfurian	1 (0.8)	5 (5)
			Moroccan & Egyptian	4 (3.3)	4 (4)
			Nigerian	5 (4.1)	1 (1)
			Beja	1 (0.8)	1 (1)
			Fonj	0	1 (1)
			Chadian	2 (1.6)	1 (1)

Frequency of DRB1 and DQB1 alleles

Our data showed that DRB1*13 (20.4%), *10 (14.2%), and *15 (12.5%) were the most frequent alleles in patients, while DRB1*13 (23.5%), *11 (22.2%), and *07 (11.7%) were in healthy controls (Table 2), as this result was previously published [17].

**Table 2:** HLA-DRB1 and -DQB1 allele frequencies in RA patients and controls and comparison of RA seropositive/seronegative ACPA. aOR (95% CI) = 0.47 (0.32-0.54); bOR (95% CI) = 2.97 (1.00-8.87); cOR (95% CI) = 0.40 (0.19-0.82); dOR (95% CI) = 0.39 (0.19-0.80); eOR (95% CI) = 1.86 (0.99-3.48); fOR (95% CI) = 3.25 (1.10-9.62); gOR (95% CI) = 0.58 (0.34-9.30); hOR (95% CI) = 3.43 (2.19-5.34); iOR (95% CI) = 0.42 (0.28-0.63). AF: allele frequency; CI: confidence interval; HLA: human leukocyte antigen; OR: odds ratio; n: number of individuals; NA: not available; RA: rheumatoid arthritis; ACPA: anti-citrullinated peptide antibodies. Correction for P-values (Pc) was calculated by multiplying the P-value with the number of alleles frequency >5% in study subjects. These data were previously published [17].

HLA-DRB1 alleles	RA patients (n = 240)		Controls (n = 196)		RA patients subgroups				Patients vs. controls		ACPA+ vs. ACPA-	
					ACPA+ (n = 63)		ACPA- (n = 57)					
	n	AF	n	AF	n	AF	n	AF	P-value	P_c_	P-value	P_c_
*01	n	AF	13	6.6	06	9.5	05	8.8	0.59		0.89	
*03	13	5.4	21	10.7	09	14.3	07	12.3	0.70		0.75	
*04	23	9.6	10	5.1	14	22.2	05	8.8	0.04^a^		0.04^b^	0.35
*07	23	9.6	23	11.7	04	6.3	05	8.8	0.01^c^	0.08	0.62	
*08	12	5.0	19	9.7	03	4.8	14	24.6	0.59		0.002^d^	0.02
*09	27	11.3	02	1.0	01	1.6	0	0	0.42		1.00	
*10	01	0.4	16	8.2	15	23.8	05	8.8	0.04^e^	0.40	0.03^f^	0.22
*11	34	14.2	24	22.2	02	3.2	07	12.3	0.65		0.06	
*13	26	10.8	46	23.5	04	6.3	05	8.8	0.44		0.62	
*14	49	20.4	0	0	0	0	0	0	0.55		NA	
*15	01	0.4	21	10.7	05	7.9	04	7.0	0.56		0.85	
*16	30	12.5	01	0.5	0	0	0	0	0.70		NA	
HLA-DQB1 alleles												
*02	36	14.8	46	23.1	15	23.4	14	24.1	0.02^g^	0.07	0.93	
*03	103	42.2	35	17.6	31	50.0	27	48.3	2.2x10^-8h^	6.6x10^-8^	0.85	
*04	02	0.8	0	0	0	0	0	0	0.30		NA	
*05	46	18.9	32	16.1	13	20.3	11	19.0	0.45		0.85	
*06	57	23.4	84	42.2	4	6.3	5	8.6	2.2x10^-6i^	6.6x10^-6^	0.74	
*07	0	0	1	0.5	0	0	0	0	0.45		NA	
*013	0	0	1	0.5	0	0	0	0	0.45		NA	

In patients, DRB1*04 and *10 were strongly associated with RA susceptibility (9.6% vs. 5.1%, P = 0.04, OR (95% CI) = 0.47 (0.32-0.54) and 14.2% vs. 8.2%, P = 0.04, OR (95% CI) = 1.86 (0.99-3.48)), with ACPA seropositivity patients (22.2% vs. 8.8%, P = 0.04, OR (95% CI) = 2.97 (1.00-8.87) and 23.8% vs. 8.8%, P = 0.03, OR (95% CI) =3.25 (1.10-9.62), respectively), and were high in frequency between the RA ethnic groups compared with the healthy controls. On the other hand, the DRB1*07 allele confers protection (5.0% vs. 11.7%, P = 0.01, OR (95% CI) = 0.40 (0.19-0.82)) and shows high frequency among healthy control ethnic groups (Tables 2, 3).

**Table 3:** The HLA-DRB1 allele frequencies among tribal groups of study subjects. Others include Nubian, Darfurian, Moroccan and Egyptian, Nigerian, Beja, Fonj, and Chadian. HLA: human leukocyte antigen; RA: rheumatoid arthritis.

Tribal groups	RA patients HLA-DRB1 (AF%)									Controls HLA-DRB1 (AF%)								
	*01	*03	*04	*07	*08	*10	*11	*13	*15	*01	*03	*04	*07	*08	*10	*11	*13	*15
Gaalia	8.3	5.8	5.0	3.3	5.0	5.0	2.5	3.3	6.7	5.1	9.2	3.1	8.2	1.0	4.1	4.1	9.2	0.0
Johayna	0.8	1.7	5.0	0.0	3.3	3.3	0.8	0.0	0.8	1.0	3.1	2.0	2.5	1.0	1.0	2.0	0.0	1.0
Baggara	0.0	4.2	1.0	0.0	3.3	3.3	0.8	0.8	0.0	3.1	3.1	0.8	1.0	1.0	0.0	1.0	4.1	0.0
Nile Nubian	0.0	0.0	3.3	0.8	1.7	2.0	1.7	1.7	0.0	1.0	1.0	0.0	2.0	1.0	1.7	1.0	0.0	1.0
Others	0.0	0.8	1.6	0.8	2.5	3.0	1.6	1.6	0.0	2.0	3.0	1.0	1.0	4.1	2.5	3.0	1.0	0.0

Inversely, DRB1*08 was significantly high in frequency in ACPA patients (24.6% vs. 4.8%, P = 0.002, (95% CI) = 0.39 (0.19-0.80)) and remained significant after multiple comparisons (Pc = 0.02) (Table 2).

In patients and as compared with the healthy controls, HLA-DQB1*03 (42.2%) and *06 (23.4%) were high in frequency, while HLA-DQB1*02 (23.1%) and *06 (42.2%) were low (Table 2).

The HLA-DQB1*03 allele was associated with RA susceptibility (42.2% vs. 17.6%, P = 2.2x10-8; OR (95% CI) = 3.43 (2.19-5.34)), and this association persisted after multiple comparisons (Pc = 6.6x10-8).

In contrast, HLA-DQB1*02 and *06 may confer protection (23.1% vs. 14.8%, P = 0.02, OR (95% CI) = 0.58 (0.34-9.30) and 42.2% vs. 23.4%, P = 2.2x10-6, OR (95% CI) = 0.42 (0.28-0.63), respectively) (Table 3). The HLA-DQB1*06 allele remained significant after multiple comparisons (Pc = 6.6x10-6).

HLA-DQB1*03 allele was high between Gaalia and Johayna tribes in patients, while HLA-DQB1*02 and *06 were higher in frequency in ethical groups of healthy controls (Table 4).

**Table 4:** HLA-DB1 allele frequencies among tribal groups of study subjects. Others include Nubian, Darfurian, Moroccan and Egyptian, Nigerian, Beja, Fonj, and Chadian. AF: allele frequency; HLA: human leukocyte antigen; RA: rheumatoid arthritis.

Tribal groups	RA patients HLA-DQB1 (AF%)				Controls HLA-DQB1 (AF%)			
	*02	*03	*05	*06	*02	*03	*05	*06
Gaalia	12.3	11.5	4.1	11.5	16.2	6.1	9.1	11.1
Johayna	3.3	3.3	0.8	3.3	3.0	2.0	4.0	2.0
Baggara	3.3	1.6	1.6	1.6	4.0	2.0	4.0	5.1
Nile Nubian	0.8	1.6	0.8	1.6	3.0	2.0	3.0	1.0
Others	4.0	1.6	0.0	1.6	6.0	4.0	6.0	5.0

Frequency of DRB1 and DQB1 haplotypes

As shown in Table 5, the HLA haplotypes DRB1*03-DQB1*03 (2.8% vs. 0.0%, P = 0.00003, OR (95% CI) = 2.54 (2.20-2.93)), DRB1*04-DQB1*03 (3.2% vs. 0.5%, P = 0.0001, OR (95% CI) = 10.85 (2.42-48.65)), DRB1*13-DQB1*02 (1.4% vs. 0.0%, P = 0.004, OR (95% CI) = 2.46 (2.14-2.83)), and DRB1*13-DQB1*03 (5.6% vs. 0.2%, P = 3.79x10-8, OR (95% CI) = 41.11 (5.48-38.46)) showed susceptibility association with RA, and each of which remained significant after correction of multiple comparison (Pc = 0.0003, Pc = 0.03, and Pc = 3.3x10-9, respectively).

**Table 5:** HLA-DRB1 and -DQB1 haplotype frequencies in RA patients and controls, and seropositive/seronegative ACPA in RA patients. aOR (95% CI) = 0.19 (0.06-0.65); bOR (95% CI) = 2.54 (2.20-2.93); cOR (95% CI) = 10.85 (2.42-48.65); dOR (95% CI) = 0.17 (0.05-0.58); eOR (95% CI) = 2.97 (1.09-8.15); fOR (95% CI) = 2.46 (2.14-2.83); gOR (95% CI) = 41.11 (5.48-38.46); hOR (95% CI) = 0.45 (0.24-0.83). HF: haplotype frequency; HLA: human leukocyte antigen; CI: confidence interval; OR: odds ratio; n: number of individuals; NA: not available; RA: rheumatoid arthritis; ACPA: anti-citrullinated peptide antibodies. Correction for P-values (Pc) was calculated by multiplying the P-value with the number of alleles frequency >5% in study subjects.

DRB1/DQB1 haplotypes	RA patients (n = 240)		Controls (n = 196)		RA patients subgroups				Patients vs. controls		ACPA+ vs. ACPA-	
					ACPA+ (n = 63)		ACPA- (n = 57)					
	n	HF	n	HF	n	HF	n	HF	P-value	P_c_	P-value	P_c_
*01/05	03	0.7	13	3.0	0	3.5	02	3.5	0.06	0.44	0.22	
*03/02	03	0.7	20	4.6	01	1.6	01	1.8	0.004^a^	0.03	1.00	
*03/03	12	2.8	0	0	05	7.9	05	8.8	0.00003^b^	0.0003	1.00	
*04/03	14	3.2	02	0.5	09	14.3	03	5.3	0.0001^c^	0.0001	0.10	
*04/06	02	0.2	05	1.2	0	0	01	1.8	0.70		0.47	
*07/02	03	1.3	22	5.1	01	1.6	02	3.5	0.002^d^	0.02	0.60	
*08/03	12	2.8	06	1.4	01	1.6	09	15.8	0.03^e^	0.22	0.01^g^	0.01
*10/05	09	2.1	12	2.8	04	6.3	02	3.5	0.92		0.68	
*11/03	09	2.1	24	5.6	02	3.2	02	3.5	0.07		1.00	
*13/02	06	1.4	0	0	0	0	03	5.3	0.004^f^	0.03	0.07	
*13/03	24	5.6	0	0.2	03	4.8	02	3.5	3.79x10^-8g^	3.3x10^-9^	1.00	
*13/06	17	3.9	45	10.4	0	0	0	0	0.01^i^	0.08	NA	
*15/06	09	2.1	21	4.9	0	0	01	1.8	0.17		0.47	

On the other hand, DRB1*03-DQB1*02 (0.7% vs. 4.6%, P = 0.004, OR (95% CI) = 0.19 (0.06-0.65)) and DRB1*07-DQB1*02 (1.3% vs. 5.1%, P = 0.002, OR (95% CI) = 0.17 (0.05-0.58)) haplotypes may confer protection against RA and remain significant following Bonferroni correction (Pc = 0.03 and Pc = 0.02, respectively). The DRB1*08-DQB1*03 haplotype was also associated with RA susceptibility (2.8% vs. 1.4%, P = 0.03, (95% CI) = 2.97 (1.09-8.15)), and ACPA seropositivity (OR (95% CI) = 0.09 (0.01-0.70), P = 0.01, Pc = 0.05), while the DRB1*13-DQB1*06 haplotype was associated with RA protection (3.9% vs. 10.4%). Both haplotypes failed to reach significance following Bonferroni correction (Pc = 0.22 and Pc = 0.08, respectively) (Table 5).

Out of the 13 haplotypes observed in the ethnic groups, nine were found in patients and healthy controls (Table 6). The shared haplotypes *03/03, 04/03*, *08/03, and *13/03 were more frequent in patients' ethnic groups, while *03/02, *07/02, and *13/06 were more frequent among the ethnic groups in healthy controls, which were found consistent with their association with RA susceptibility and protection, respectively (Table 6). The RA-susceptible *03/03 and *13/03 haplotypes were absent in the healthy ethnic groups, while the protective *13/06 haplotype was absent in the patient ethnic groups.

**Table 6:** The HLA-DRB1 and -DQB1 haplotype frequencies among tribal groups of study subjects. HF: haplotype frequency; HLA: human leukocyte antigen; RA: rheumatoid arthritis.

DRB1/DQB1 haplotypes	RA patients tribal groups (HF%)					Controls tribal groups (HF%)				
	Gaalia	Johayna	Baggara	Nile Nubian	Others	Gaalia	Johayna	Baggara	Nile Nubian	Others
*03/03	3.3	1.7	2.5	0.0	0.8	0.0	0.0	0.0	0.0	0.0
*04/03	2.5	4.2	0.8	2.5	0.0	1.0	0.0	0.0	0.0	0.0
*08/03	2.5	2.5	2.5	0.0	0.8	1.0	0.0	1.0	1.0	0.0
*10/05	2.5	0.8	1.7	0.0	0.0	4.1	1.0	2.0	2.1	1.0
*13/03	1.7	0.0	0.8	1.7	0.0	0.0	0.0	0.0	0.0	0.0
*13/06	0.0	0.0	0.0	0.0	0.0	9.3	0.0	4.1	0.0	0.0
*03/02	0.8	0.0	0.8	0.0	0.0	8.2	3.1	3.1	1.0	0.0
*01/05	1.7	0.0	0.0	0.0	0.0	5.2	1.0	3.1	1.0	1.0
*07/02	0.8	0.8	0.0	0.8	0.0	7.2	1.0	1.0	2.1	1.0

## Discussion

The HLA genes consist of three main subregions (DP, DQ, and DR), which code HLA class II molecules important in antigen presentation, particularly to immune cells that are involved in adaptive immune activation via T cell activation and differentiation. The polymorphisms in the HLA class II molecules account for approximately 30-40% of the genetic risk for RA [18]. Among the HLA-linked genes, many studies have reported that polymorphisms at the HLA-DRB1 locus confer more risk for RA and other autoimmune diseases [19]. Thus, particular HLA-DRB1 alleles like HLA-DRB1*01, HLA-DRB1*04, and HLA-DRB1*10 were found to be associated with the development of RA in ethnic groups from Europe and Asia [20-23]. On the other hand, certain HLA-QB1 alleles, such as HLA-QB1*05, *04, and *602, were also found to be associated with RA risk in populations from Asia [22,24] and Africa [25].

We reported the high frequency of HLA-DRB1*04 and *10 alleles in RA patients compared with healthy controls [17]. In this study, the HLA-DRB1*04 and *10 allele frequencies were remarkably high between the RA ethical groups, which is in concordance with population studies from different races [13,26-32]. Furthermore, associations were reported between the HLA-DRB1*10 and RA risk in populations from Senegal, Gambia, and Cameroon, without significant differences between their ethnic groups [33,34].

Another study from the USA showed the HLA-DRB1*04 frequency was higher among non-Hispanic white and Hispanic ethnic groups compared with African Americans [33]. African Americans were characterized by a low prevalence of RA due to the low frequency of the high-risk alleles HLA-DRB1*04:01 and *04:04 [8]. Collectively, these studies indicate that the HLA antigens are associated with RA susceptibility among ethnic groups.

Furthermore, HLA-DRB1*04 and *10 alleles were statistically associated with ACPA seropositivity [17]. This association was reported in other studies [14,34-37]. Likewise, HLA-DRB1-SE, particularly the HLA-DRB1*04:05 allele, was found to be significantly associated with RA risk in ACCP-positive patients from Egypt [38] and Saudi Arabia [39]. Moreover, strong evidence suggests that HLA-DRB1*01, *04, and *10 behave as susceptible alleles, especially for ACPA seropositivity [40,41].

We found that HLA-DRB1*07 had a higher frequency in healthy control ethnic groups than in RA patients and may confer protection. This protective effect has been observed in different populations [42-47]. While other studies found an equal distribution of the allele between the patients and controls [21,27].

Our data showed that HLA-DQB1*03 frequency was significantly higher in patients compared with the healthy controls, with an inconsistent frequency between ethnicities. This result agreed with an ethnic group from Iran [28]. On the contrary, HLA-DRQB1*02 and HLA-DRQB1*06 were significantly higher in healthy controls.

Interestingly, the aforementioned two alleles showed protective associations in populations from Albania [48], susceptibility associations in populations from Iraq [49], and no associations in populations from Turkey [47].

In the present study, several haplotypes were found to be associated with RA risk, including HLA-DRB1*04/DRB1*03, HLA-DRB1*08/DRB1*3, HLA-DRB1*03/DRB1*03, HLA-DRB1*13/DRB1*03, and HLA-DRB1*13/DRB1*02. Similarly, susceptibility to RA was reported in patients with HLA-DRB1*03/DRB1*03, while HLA-DRB1*13/DRB1*03 showed a protective association [50].

Collectively, the association between HLA genotyping and inheritable makeup is still controversial due to the differences in race between populations.

We demonstrated a protective association of HLA-DRB1*03/DRB1*02, HLA-DRB1*13/DRB1*06, and HLA-DRB1*07/DRB1*02 haplotypes with RA. The HLA-DRB1*07/DRB1*02 haplotype protective association has been previously stated in a population from Iraq [49]. In resemblance, a study conducted among the Moroccan population states that DRB1*13-DQB1*06 and DRB1*07-DQB1*02 haplotypes are more frequent in RA patients positive for RF as compared with healthy controls [51]. Likewise, the HLA-DRB1*03/DRB1*02 protective association has been reported in patients with type 1 diabetes [52] and celiac disease [53]. The differences in RA risk and protective alleles between our data and others could be explained by the differences in the prevalence of RA between populations, while the effects of genetic and environmental factors cannot be excluded.

The present study has some limitations. The small sample size, the expensive cost of HLA typing tests, and the wide discrepancy in Sudanese ethnic groups lead to a lack of association of allele frequencies between the tribal stock. More hands-on studies with large sample sizes and further studies using high-resolution genotyping are needed to verify our findings.

## Conclusions

This study highlighted the relevance between the ethical background (i.e., tribal stock) of the Sudanese population, the HLA class II molecules, and RA risk that can be used as a foundation for future population-based assessments. Our data identified nine HLA-DRB1 and four DQB1 alleles among 11 ethnic groups and their modest consistency with RA susceptibility, despite their modest differences in frequency. Further studies with a large sample size are needed to prove these associations.
